# Anthropogenic mortality threatens the survival of Canarian houbara bustards

**DOI:** 10.1038/s41598-024-52641-z

**Published:** 2024-01-24

**Authors:** Juan C. Alonso, Inmaculada Abril-Colón, Alberto Ucero, Carlos Palacín

**Affiliations:** Department of Evolutionary Ecology, Museo Nacional de Ciencias Naturales (CSIC), José Gutiérrez Abascal 2, 28006 Madrid, Spain

**Keywords:** Ecology, Zoology

## Abstract

Anthropogenic mortality is a major cause of global mortality in terrestrial vertebrates. Quantifying its impact on the dynamics of threatened species is essential to improve their conservation. We investigated cause-specific mortality in Canarian houbara bustards (*Chlamydotis undulata fuertaventurae*), an endangered subspecies endemic to the Canary Islands. We monitored 51 individuals tagged with solar-powered GSM/GPRS loggers for an average of 3.15 years, and recorded 7 casualties at aerial lines (13.73% of the sample; 5 at power lines, 2 at telephone lines), 1 (1.96%) at a wire fence, 4 road kills (7.84%) and 1 case of predation by cat (1.96%). Cox proportional hazards models showed that anthropogenic and natural annual mortality rates were similar (respectively, 6.20% and 6.36% of the individuals). We estimate that 33–35 houbaras die each year in the Canary Islands due to anthropogenic causes. Population viability models using these data and juvenile productivity values obtained over seven years predicted the extinction of the species in 50 years. Eliminating anthropogenic mortality, the population could be recovered, but would still require management actions to improve habitat quality. Conservation measures to reduce anthropogenic mortality due to power line fatalities, roadkills and predation by cats, as well as to increase productivity, are urgently needed, particularly on Fuerteventura, where houbaras are on the brink of extinction.

## Introduction

Quantifying mortality and establishing its causes is essential for understanding the dynamics of animal populations^1^, yet this has always been difficult, as it can only be assessed using tagged individuals^2^. Besides mortality due to natural causes, it is important to determine mortality due to human activities, in order to establish sustainable conservation programs^3,4^. Predation by domestic or feral cats *Felis silvestris catus*, road-kills and collisions with power lines are among the causes of anthropogenic mortality with the greatest impact on vertebrate species^5–10^. As an example, Loss et al.^7^ estimated that these two factors represented respectively the first, third and fourth leading causes of bird mortality in the USA and Canada, with annual losses of respectively more than 5000, 200, and almost 50 million individuals. In Spain, collision with power lines seems to be the main cause of bird mortality, with 31% of total records (n = 162,127) in the period 2008–2018^11^, although predation by cats has not yet been adequately quantified^12–14^. Recent reviews concluded that anthropogenic mortality accounts for a quarter of global mortality in terrestrial vertebrates^8^, and that the rate of vertebrate species loss over the last century is up to 100 times higher than the rate prevailing between previous mass extinctions^15^. Moreover, increases in global road network and electricity demand expected by 2050 (respectively, 14–23%^16^, and ca. 50%^17^) will enhance their negative impacts on biodiversity, and probably lead to the extinction of some of the most vulnerable species, unless effective mitigation measures are developed and implemented^18–21^.

Certain groups of birds, such as cranes and bustards, are especially vulnerable to collision with power lines^22–30^, as they have less maneuverability in flight due to their large size and reduced frontal visibility compared to other birds^31,32^. Specifically bustards were identified among the most vulnerable species based on fatality risk indices using various morphometric and habitat parameters^33,34^. For example, in little bustards (*Tetrax tetrax*) and great bustards (*Otis tarda*), fatalities at power lines reach critical annual mortality rates (respectively, 3.4–3.8%^28^; 3.5–6.6%, with extreme values of up to 6–13% in some populations^35–37^). In a great bustard population in central Spain, collision with power lines was found to be the major cause of non-natural mortality (37.6–55.0%^38,39^). In the same region, transport power lines (> 50 kV) that intersect with great bustard home ranges cause up to 2.46 collision fatalities per km and year^40^. Adding power line fatalities to natural mortality had a significant negative effect on population viability in this^37^ and other populations of this species^35,41^. As an example, the disappearance of a great bustard lek in central Spain due to the death of at least 29 individuals at a single power line was documented in detail between 1996 and 2003^42^. Negative consequences of power line fatalities have been also described for other medium or large-sized bustards in Asia and Africa^23,43–47^. Although marking cables with anti-collision devices has been practiced over the last decades as a supposedly effective mitigation measure^27,48,49^, its effectiveness is quite variable depending on design^20^, and collision mortality cannot be completely eliminated^24,30,50^. Therefore, for highly threatened species this measure is not sufficient and specifically for bustards alternative mitigation measures are urgently required^51^, with power line undergrounding apparently being so far the only effective method^52^. Finally, in the case of species that fly or migrate at night, such as cranes, recent studies have shown that ultraviolet avian collision avoidance systems, glow-in-the-dark devices, and other types of nocturnal specific flight diverters can reduce collisions considerably^53–55^.

As for road-kills, among vertebrates it affects proportionally more amphibians, reptiles and small to medium-sized mammals, but also birds, particularly ground-dwelling species, and there are no mitigation measures that are effective with all animals, so vehicle collision effects on endangered species can be very detrimental^9,56–58^. In sum, although the problem of anthropogenic mortality is well known, there is still insufficient data on its real impact on many species, and experts are calling for additional research to provide more specific data on true mortality rates especially for the most threatened taxa, in order to correctly assess its demographic consequences and design effective mitigation measures^11^.

In this study we quantified mortality in the Canarian houbara bustard (*Chlamydotis undulata fuertaventurae*), using GSM/GPRS telemetry. Our objective was to identify the main anthropogenic mortality causes in this globally threatened species, assess their impact compared to natural mortality, and predict demographic trends under two scenarios, natural mortality only, and both natural and anthropogenic mortality. Previous studies based on carcass counts drew attention to the importance of power line and vehicle collision mortality in Canarian houbaras^59–64^. However, carcass counts do not allow an accurate, unbiased quantification of mortality rates, because data need to be transformed to account for various sources of error in carcass detectability, and later extrapolated to the total distribution area of the species, which may lead to additional errors^65–72^. The method used here was based on a daily monitoring of the fate of tracked individuals over several years, and thus provided accurate mortality estimates from both, natural and anthropogenic sources^73–75^, which should help to design appropriate management measures to mitigate its effects on the population dynamics of this endangered species.

## Results

We obtained survival data from 51 houbara bustards (30 males, 21 females) from the date when they were captured (18th January 2017 to 26th January 2021) until January 1st, 2023. The birds were monitored for an average of 3.15 years (range = 46–1837 days). Over that period, we recorded 7 casualties at aerial lines (13.73% of the sample; 5 at power lines, 2 at telephone lines), 1 (1.96%) at a wire fence, 4 road kills (7.84%) and 1 case of predation by cat (1.96%) (Table 1). The total number of deaths due to these three anthropogenic causes was 13 (25.49% of the sample; 9 males, 4 females), compared to 8 deaths due to natural causes (15.69% of the sample; 5 males, 3 females) (Table 1). The breakdown by sex indicates that 10.0% (3/30) of males and 19.0% (4/21) of females were due to collisions with aerial lines. As for wire fence collisions, road-kills and cat predation, respectively 3.3% (1/30), 13.3% (4/30) and 3.3% (1/30) of males were killed, but no females were affected (Table 1).

**Table 1:** Number of houbara bustards by sex and cause of mortality (anthropogenic, natural).

Sex	Anthropogenic mortality					Natural mortality	Total birds tagged
	Collision with			Road kill	Cat predation		
	Power line^a^	Telephone line	Wire fence				
Males	2	1	1	4	1	5	30
Females	3	1	0	0	0	3	21
Total	5	2	1	4	1	8	51

Data from the Canary Islands (Lanzarote, Fuerteventura, La Graciosa), 2017–2023.

^a^These 5 casualties occurred at distribution lines of 15-20 kV without anti-collision devices.

**Figure 1:**
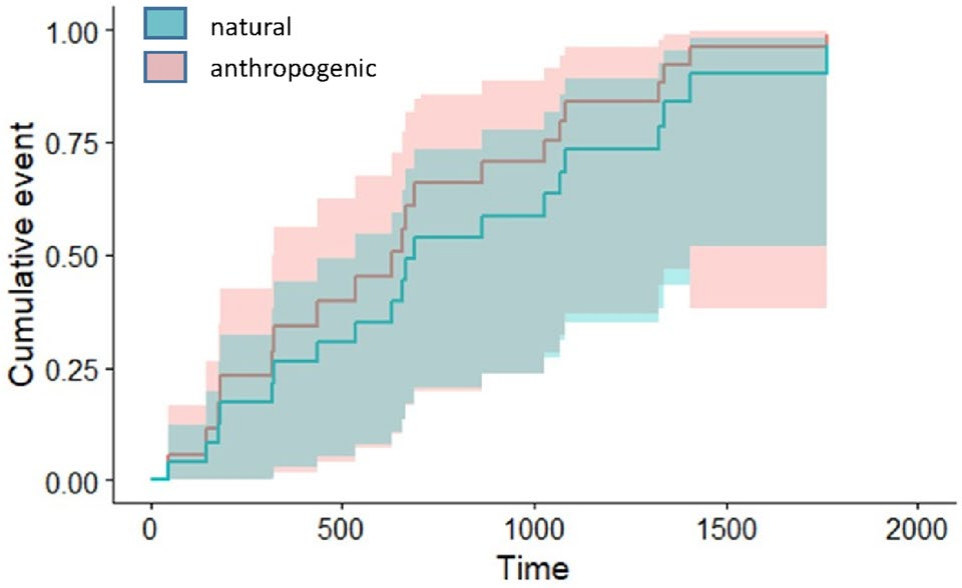
Cumulative incidence function for houbara bustard mortality cause (anthropogenic *vs* natural) in the Canary Islands. Time is given in days.

The relative risks of anthropogenic and natural mortality derived from the Cox model were similar, with a slightly but not significantly higher hazard ratio for natural mortality (Table 2, Fig. 1). Estimated anthropogenic and natural annual mortality rates were respectively 6.20% and 6.36% of individuals in the population. The most plausible model also showed a smaller hazard ratio in sedentary birds compared to migratory birds, but the difference was not significant (Table 2). To further examine mortality risk associated to migration we looked in more detail at collisions with aerial lines, which is the largest anthropogenic mortality source, and could reasonably affect migratory individuals to a greater extent, since they perform long flights between their breeding and post-breeding areas twice a year. We found that all seven aerial line casualties (Table 1) involved migratory individuals, which suggests a greater vulnerability of migrants to aerial line collision (p = 0.034, Fisher's exact probability test). In four of these seven cases (2 males, 2 females), the collision occurred during a true migratory flight between the breeding and the post-breeding areas, and in two other cases (1 male, 1 female), it happened during a flight between disjunct zones within the post-breeding area, one of them a nocturnal flight. Thus, six of the seven fatal accidents (85.7%; 3 males and 3 females) occurred on migration-related flights, whereas only in one case (14.3%) the accident occurred during a diurnal flight in the breeding area. This case, together with one of the two fatalities in the post-breeding area, were the only two fatalities occurring by day; most fatal collisions (5 out of 7, i.e. 71.4%) occurred at night (respectively, at 23:15 h, 23:25 h, 0:00 h, 0:05 h, and one case at undetermined exact time, but certainly at night, as it took place between 21:00 and 05:00 h in November).

**Table 2:** Output of the Cox proportional hazards model describing mortality of houbara bustards in the Canary Islands, considering anthropogenic and natural mortality risk.

Variable	Coefficient	Hazard ratio	SE	Wald	P	95% CI
Cause of death (natural)	0.133	1.142	0.528	0.253	0.801	0.406—3.213
Migratory status (sedentary)	−0.374	0.688	0.513	−0.729	0.466	0.252—1.880

The best model selected through AIC_c_ included mortality cause (anthropogenic vs natural) and migratory status (migratory vs sedentary). AIC_c_ = 82.759, W_i_ = 0.798, df = 2.

As for differences between males and females, sex was retained in the second most plausible model (AIC_c_ = 85.581, df = 3), together with cause of death and migratory status (males: coefficient = 0.112, hazard ratio = 1.118, SE = 0.657, p = 0.865). Although sex differences were not significant, males showed 4.2% lower annual survival values than females (Supplementary Table S2 online).

**Figure 2:**
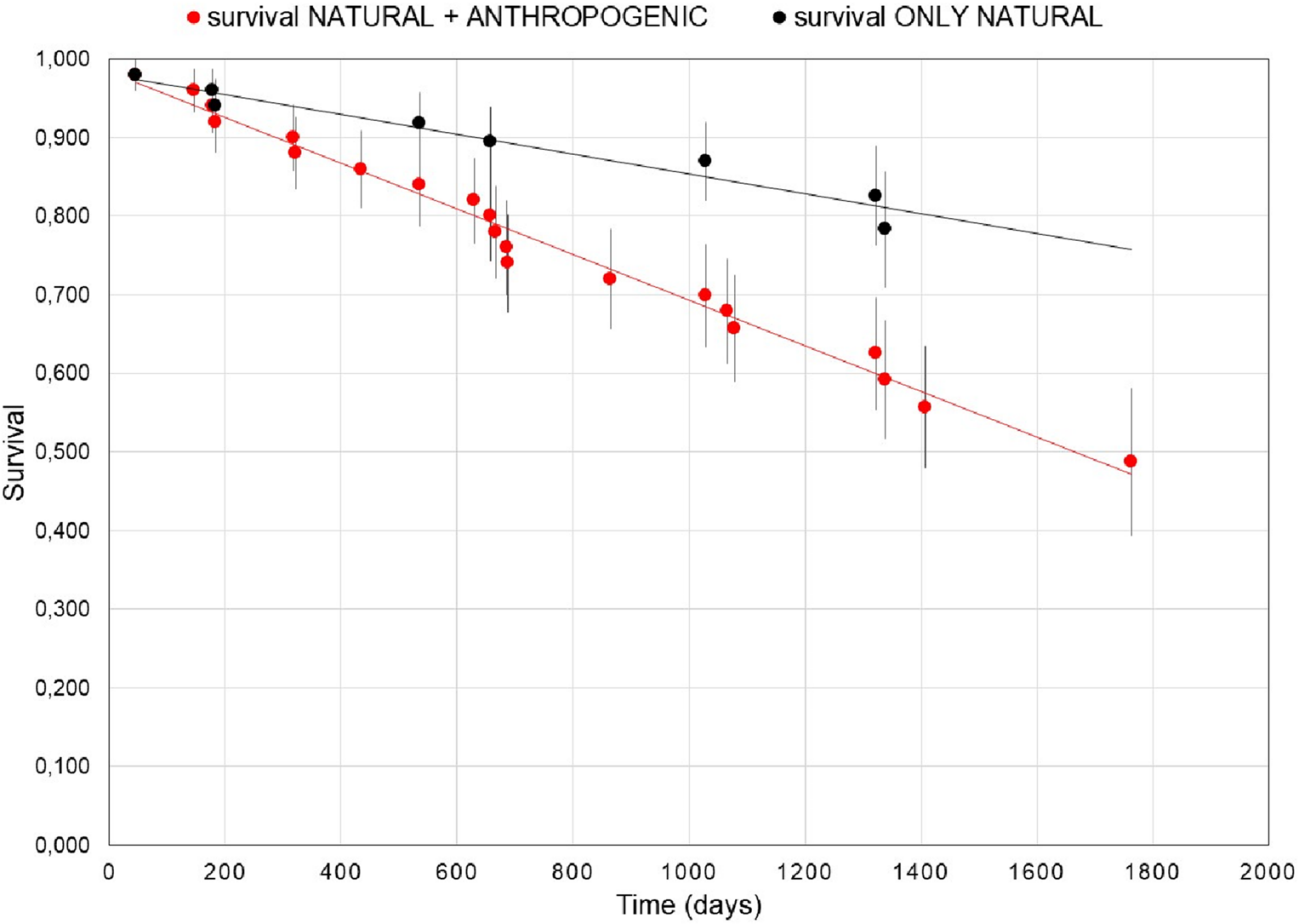
Survival probability functions of houbara bustards in the Canary Islands obtained using ‘survfit’ of the ‘survival’ package^124^ and assuming two scenarios: all causes of mortality operating (i.e., natural and anthropogenic, red dots) and only natural mortality (i.e., anthropogenic sources suppressed, black dots). Survival estimates and standard errors are given. See Supplementary Table S2 for details.

Figure 2 shows the survival functions based on the Cox model including all birds (i.e., without covariates for sex and migratory status) for two scenarios: the real one, in which all causes of natural and anthropogenic mortality are operating, and a natural mortality only scenario, in which anthropogenic mortality is suppressed. The respective annual survival rates were 0.874 for the scenario with natural and anthropogenic mortality, and 0.936 for the natural mortality only scenario. Comparing both functions, we estimated that a proportion of 0.062 (6.20% individuals) per year is attributable to anthropogenic causes, compared to 6.36% due to natural causes (Fig. 2, Supplementary Table S3 online).

Taking into account the houbara population sizes estimated in each island, and the fact that there are no aerial lines and asphalt roads in La Graciosa, these numbers imply that each year some 33–35 houbaras would die from anthropogenic causes in the Canary Islands (Supplementary Table S4 online).

**Figure 3:**
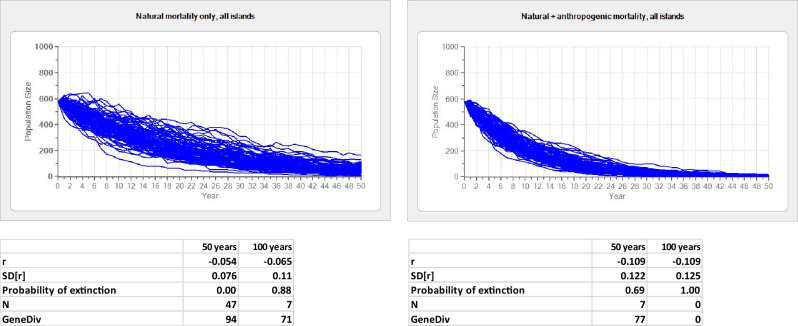
Simulated trajectories of the houbara bustard population of the Canary Islands (n = 577 individuals) over 50 years under two scenarios, natural mortality only or both natural and anthropogenic mortality. For each simulation, the exponential rate of increase (mean and SD), probability of extinction, final population size and mean genetic diversity (or expected heterozygosity) remaining in the extant population are given. The values for an extended simulation period of 100 years are also given. The mean time to first extinction under natural mortality only was 44.32 years (SD = 4.27). Supplementary Fig. S1 online shows simulations run independently for each of the two main islands (Lanzarote, Fuerteventura).

All simulated population trajectories showed negative trends for both scenarios, natural mortality only and both mortality sources (Fig. 3, Supplementary Figure S1 online). However, when anthropogenic mortality was included, negative rates of increase doubled, accelerating the extinction process, and exceeding 50% extinction probability over 50 years in all cases, either taking the entire houbara population of the archipelago as initial size (Fig. 3), or separately the population estimates in both major islands (Lanzarote and Fuerteventura; Supplementary Figure S1 online). In a scenario of only natural mortality, the entire population survived a 50-year period, yet with a significant population reduction, but also showed a high extinction probability at 100 years, when a final population of only seven birds was predicted (Fig. 3). The most dramatic prediction was that in Fuerteventura, where the particularly low breeding success of the species does not help to sustain the population, even in a scenario of only natural mortality (Supplementary Figure S1 online).

## Discussion

We found that anthropogenic mortality was considerable, affecting 6.2% of Canarian houbara bustards each year. This value almost equaled that of natural mortality, and the sum of both (annually, 12.56% of individuals) exceeds the mortality rate that would be demographically sustainable, considering that the average annual productivity is only 7.15% (Supplementary Table S1 online, footnote). Indeed, all population trajectories simulated under a scenario of natural plus anthropogenic mortality predicted the extinction of the species. Even suppressing anthropogenic mortality, Canarian houbaras are subject to an 88% probability of extinction within 100 years, although it is true that in the first 50 years their survival would be guaranteed, yet with a very drastic population reduction. Simulations are particularly worrying on Fuerteventura, where the probability of extinction is 100% under any scenario, either the current one with all mortality sources operating, or one of only natural mortality. These results warn of the serious risk that anthropogenic mortality represents for the short-term survival of houbaras in that island, and in the long term even in the whole archipelago, if no management and conservation measures are urgently implemented. The results presented here support the idea that human-induced mortality could have been a major cause of the remarkable population decline observed during the last decades in Fuerteventura, where the species is currently on the brink of extinction^76–79^. Anthropogenic mortality could have also contributed to the apparent shift from an increasing to a decreasing demographic trend only a decade ago suggested recently for Lanzarote^80^, the island that today represents the main stronghold of this endangered species. Our study adds new evidence from a globally threatened species on the importance of anthropogenic mortality as a major cause of vertebrate population decline^4,7,8^.

The extinction prediction under a natural mortality-only scenario was unexpected and raises the question as to whether survival or reproductive rates might have decreased in recent years. We actually suspect that these rates could have been higher in the past, before the period of climate aridification that started around 1980 in our study area, which led to an increase in temperatures compared to the 1961–1990 climatological baseline period^81,82^. In the Canary Islands, we have observed the negative influence of the absence of rainfall on the juvenile productivity of houbara bustards (own unpublished data). Also in Morocco, temperature increases and more severe droughts have been documented since 2010^83,84^, which would explain the reduced survival of houbara bustards in that country in recent years^85^. In semi-desert habitats, changes in temperature and rainfall may have a major impact on primary production and thus on food resource availability and breeding performance of birds^86–90^. Another possibility is that annual productivity might have been underestimated in our models because some females with dependent young remain several months in their remote breeding territories, some of which are not normally accessed in productivity surveys. We had two such cases out of 14 tagged females in 2023. However, the fact that the productivity of tagged females was very close to that measured in population surveys would support that the population value we measured would not be significantly underestimated. To assess the importance of productivity in our population viability simulations, we estimated the minimum proportion of successfully breeding females required to maintain a stable population. This value was found to be 23% if all causes of anthropogenic mortality were removed. Otherwise, if these anthropogenic mortality causes were to continue to operate as they do today, the percentage of females breeding successfully would have to rise to 32%; at 23% the population would not go extinct in 50 years, although it would be drastically reduced (Supplementary Figure S2 online). To achieve this increase in female reproductive success, very important habitat management measures would be necessary, especially in view of the aridification of the islands' climate in recent years.

In sum, we believe that the successfully breeding females that may have gone unnoticed in remote breeding areas are not sufficient to raise the productivity value to the magnitude reflected in Supplementary Fig. S2. To a large extent, the low reproductive success of Canarian houbaras has been due to the lack of sufficient rainfall in recent years. Suppressing the influence of recent droughts, juvenile productivity would probably be higher and model predictions would certainly be less pessimistic in a natural mortality-only scenario.

Collisions with aerial lines had the highest incidence among human-induced mortality sources evaluated, with almost twice as many deaths as road kills. Although migratory status had no significant effect in the model, a probability test comparing aerial line casualties between migratory and sedentary birds suggested a greater vulnerability in migratory individuals. This seems reasonable considering that houbaras typically migrate by night, when cables are not visible^91^. Our sample is small to draw definitive conclusions, but we find it noteworthy that 71% of the collisions occurred at night, most of them during migratory flights. If future studies confirm a higher collision rate in migratory individuals, this could lead to a progressive decrease in the migratory fraction of the population, unless mortality due to other causes is higher in sedentary individuals, compensating for this imbalance in the mortalities of both fractions. For example, food availability decreases in most reproductive areas due to summer drought^91^, which could affect survival of sedentary birds remaining at their breeding areas. Alterations in the balance of population fractions by anthropogenic mortality is not rare and has been documented previously in several species. A study with great bustards showed that migratory males suffered higher mortality due to power line casualties than sedentary males, and suggested that this had caused a decrease in the migratory fraction of the population over a sixteen-year period^39^. Even the normal sex ratios of some species have been altered by differential human-induced mortalities among sexes^92–94^.

Our results about possible sex differences in mortality also deserve some discussion. Males showed lower annual survival rates than females, and although the difference did not reach statistical significance in hazards models, it was far from negligible (4.2%) and consistent among scenarios (survival under natural mortality only: 0.931 in males *vs* 0.952 in females; survival under anthropogenic mortality only: 0.927 in males *vs* 0.950 in females). A higher mortality in males is in accordance with the female-biased sex ratio observed in the population (ca. 1.4 females per male in Lanzarote^95^; similar values for Fuerteventura; own unpubl. data). Previous studies did not find significant sex differences in mortality of wild-born houbara, but found male survival to be lower than female survival among captive-bred released birds^85^. Higher mortality rates in males have also been reported for the great bustard, a species that also shows a female-biased sex ratio^37,38,96^. A higher male mortality is expected in polygynous species with male-biased sexual size dimorphism favoured by sexual selection^97,98^, and this could also be the case in houbara bustards, which have a mating system that fits the definition of dispersed lek, intense male sexual display, and a male-biased sexual size dimorphism^99–101^.

Regarding sex differences in anthropogenic mortality, one would expect more males to be killed by collisions with power lines, due to their larger size and less maneuverability in flight. However, our sample of tracked females included a higher proportion of migratory individuals (85.7%; 18 out of 21), compared to what is normal in the population (ca. 35% migrants in both sexes; Abril-Colón et al. 2022). This probably contributed to our finding of almost twice as many collisions in females (19% of 21 tracked females) than in males (10% of 30 tracked males). In addition, our data showed that females still have a higher tendency to perform local, non-migratory flights within their home ranges than males (respectively, 0.0024 flights/h, SE = 0.0012, and 0.0016, SE = 0.0007; p < 0.005, Mann–Whitney test; sample of 3433 local flights, i.e. after excluding migratory flights; own unpubl. data). This higher tendency to fly in females, which has also been observed in great bustards (pers. obs.), makes them more vulnerable to collisions with power lines than males, and would also contribute to explain why we found more power line fatalities in females.

In contrast, it is noteworthy that the victims of the four collisions with vehicles were males. It is tempting to speculate that males could be more vulnerable to road kills because during their display runs to attract females or chase competitors they naturally take more risks due to their aggressiveness and may pay less attention to vehicles, or perhaps have their view largely obstructed by the display feathers. All four road kills involved sedentary males, and three of them indeed occurred between end of October and late December, when males are very active defending their territory. Tejera et al.^63^ identified vehicle collisions as an important mortality source in Canarian houbaras. They guessed similar road kill rates (seven casualties estimated from two individuals found, both in spring, when houbaras are still in their territories), but unfortunately the birds were not sexed. Our sample is still small to draw definitive conclusions, but current results would provisionally suggest that males could be more vulnerable to road-kills and females to collisions with power lines, perhaps due to their respective greater propensities to run and to fly.

As for the incidence of anthropogenic mortality in similar species, in the little bustard it was also found to reach similar values to natural mortality (respectively, 7.4% and 8.7% annual rates^28^), with power line fatalities being the main cause of mortality (3.4–3.8%/year), followed by poaching (2.4–3%/year), though the overall annual survival of little bustards was considerably lower than that found in Canarian houbaras (66.5%^28^). Human-induced mortality was much higher in migratory populations of the MacQueen’s bustard, where hunting and poaching represented a major fraction of the annual mortality rate (28.3%^102^; 19.7%^45,103^), though up to 30.7% summer deaths were attributed to power line collisions^45^. Annual survival rates found in Canarian houbaras were similar to those reported for non-hunted populations of African houbaras (75–86%^104^, 80–90%^105^) and MacQueen’s bustards (80.3% excluding hunting losses^103^).

For the first time we used GSM/GPRS telemetry in Canarian houbara bustards as an accurate method to estimate survival^73–75^. Previous studies based mortality estimates on carcass searches along power lines or roads, but this method does not allow a direct calculation of annual mortality rates because the sample size to which to assign deaths is unknown. Even after applying correction factors to account for effects of scavengers, deaths far from the line, and observer experience, the results of carcass searches may lead to inaccurate mortality estimates^65–72^. For example, Lorenzo and Ginovés (^61^, Table 11), after establishing a percentage of carcass loss by scavengers, estimated a total of 70 dead houbara bustards per year in Lanzarote and 164 in Fuerteventura. Applying all correction factors mentioned above, the authors raised their estimate to 116 per year in Lanzarote and 286 per year in Fuerteventura. Considering population sizes in both islands (440–452 in Lanzarote^95^; 85–109 in Fuerteventura^78^), these authors’ estimates would imply annual mortality rates of 25.7–26.4% for Lanzarote and 262.4–336.5% for Fuerteventura. Using Lorenzo & Ginovés’s population estimates^61^, which were higher than ours, mortality estimates were somewhat lower (10.4% in Lanzarote and 14.1% in Fuerteventura without correction factors, and about 60% in both islands applying correction factors;^61^, p. 39). Another study estimated annual mortality rates of 25.5% for the entire houbara population on both islands^64^. All these mortality rates are obviously overestimates, since if true, they would have definitely led to the extinction of Canarian houbaras in only a few years.

### Conclusions and conservation recommendations

In conclusion, our study provided for the first time reliable mortality estimates for Canarian houbara bustards, and insights into the potential effects of anthropogenic mortality on their survival probability. Due to logical limitations in capture and tagging permits in a threatened species with a low population size, our sample size was admittedly small to achieve the desirable power in some of the statistical analyses. However, tracked birds represented close to 10% of the total population, and the results shown were sufficient to assess the importance of human-induced mortality on a bird threatened with extinction. The high mortality rates due to collision with aerial lines and road kills require the immediate adoption of corrective measures. In particular, in the main core areas of houbaras, (a) all telephone lines and at least the most dangerous sectors of power lines should be buried. If undergrounding of power lines is delayed for cost reasons, these sectors should be marked, preferably with anti-collision devices that have some effectiveness at night, when most houbaras migrate. Research on nocturnal specific flight diverters should be carried out to find the best cost-effective option to mitigate nocturnal collisions of Canarian houbaras. (b) Corrective measures to prevent roadkills, such as speed limits controlled by radar or speed bumps should be implemented. (c) Wire fences should be removed or adequately signalled. (d) The numbers of unowned and owned free-roaming domestic cats and dogs should be reduced. In addition to these actions to mitigate anthropogenic mortality, habitat management measures should also be implemented. Specifically, irrigated legume fields should be planted as supplementary food during summer, the most critical season for Canarian houbaras, and *Launaea* shrub vegetation should be regenerated to improve habitat quality at certain breeding areas in an attempt to improve juvenile productivity, which is currently below the limit that allows the species to be viable in the archipelago.

## Methods

### Study area and species

The study was carried out in Lanzarote, Fuerteventura and La Graciosa, the three easternmost islands of the Canary archipelago (respectively, 29°02′, 13°37′ W, 986 km^2^; 28°24′, 14°00′ W, 1660 km^2^; 29°15′ N 13°30′ W, 29 km^2^). The Canary archipelago is located 140 km west of the northwestern coast of Africa, in the Atlantic Ocean. The subtropical-desert climate of the three islands is tempered by the cold Canary Current and the almost permanent northeasterly trade winds. The mean annual temperature is 21 °C, and the average annual rainfall is around 100–120 mm, concentrated in December-February, and with higher values in La Graciosa and Lanzarote than in Fuerteventura, which is the most arid island of the archipelago. Summers are dry, with less than 1 mm precipitation in June–August. The islands have a volcanic origin, and the vegetation is characterized by xerophytic shrubs, modified in some areas by goat grazing and farming activities. The basis of the economy today is tourism, which has replaced the agricultural and livestock economy of past centuries. These islands now represent major tourist destinations and their natural habitats, including many protected areas and endemic species of flora and fauna, are threatened by a potentially unsustainable development.

The Canarian houbara is a subspecies of African houbara bustard endemic to the three islands, classified as globally vulnerable according to IUCN criteria^106^, and as endangered in the Spanish Red List of Birds^78^. Canarian houbaras inhabit all three islands, with their main stronghold in Lanzarote (440–452 birds), and smaller populations in Fuerteventura (85–109 birds) and La Graciosa (12–16 birds)^78,95^. The species is polygynous^107,108^ and exhibits an exploded lek mating system^99,100^. It shows a moderate male-biased sexual size dimorphism^101^. The Canarian subspecies is a nocturnal and partial migrant^91^. Over one third of the individuals abandon their breeding areas and migrate to non-breeding areas with a mosaic of shrubland and cropland where they spend the hottest and driest months of the year. Between late autumn and early spring, males concentrate at lek areas where they display at specific locations of their territories, to which they generally remain faithful over the whole breeding period and also between years^83^. These sites are characterised by a high visibility and low stone and vegetation cover, and are far from human infrastructure^109^. Females visit displaying males for mating and later take over all breeding duties^110^. Successfully breeding females normally raise one, less frequently two or three chicks that remain dependent of her for several months. Females with dependent chicks abandon their breeding area later than unsuccessful females^91^. Adult Canarian houbaras have no natural predators, except feral or free-ranging dogs or cats, and Canarian ravens (*Corvus corax canariensis*), Yellow-legged gulls (*Larus michahellis atlantis*), and Common Buzzard (*Buteo buteo insularum*), which prey on eggs and chicks and may even attack injured or debilitated adults^78^.

### Data collection

We used data from 51 houbara bustards (30 males, 21 females) captured with nylon snares and equipped with backpack-mounted, solar-powered GSM/GPRS loggers (25 g model for females, 48 g model for males; e-obs GmbH, Gruenwald, Germany). We used an elastic band as harness material to minimise friction with skin and contour plumage. The weight of transmitter plus harness was on average 2.15% of the body weight in females (range 1.81–2.53) and 2.83% in males (range 2.54–3.15). We captured males at display sites and females at foraging areas during the non-breeding season, in order not to put at risk their nesting process. The capture team consisted of four people, who remained close to the capture site and accessed as soon as birds were caught in the snares. Birds were immobilized and their heads covered during the marking process to minimize capture stress. The average processing time was only 14 min (range 5–25) and we did not observe behavioural alterations after release. Sex was established in the field using distinctive plumage features^111,112^, and confirmed by genetic analysis using DNA extracted from 1–2 contour feathers plucked from each bird^113^. Only three birds (one female, two males) showed plumage and biometric characters that made us suspect they could be immatures, all other birds were identified as adults^101^.

The loggers recorded GPS locations at five-minute intervals continuously throughout 24 h from September 2020; before that date, GPS locations were recorded between 05:00 and 22:59 UTC, i.e. from 1 (summer solstice) to 3 h (winter solstice) before sunrise to 1 to 3 h after sunset. The loggers were programmed to record one GPS location every five minutes when the charge level was high, which in our study area was usual due to the prevailing sunshine, and every 30 min otherwise. All loggers were provided with an accelerometer (ACC) that registered the acceleration of the bird on a three-dimensional space, providing a 3D-graph representation of the bird's movements. Combining GPS locations and ACC pattern enabled us to know the behaviour of the bird immediately before collision, the time of collision, and in most cases, the time and route of the last movement, either flying or walking, and so facilitated the diagnosis of the cause of death. In cases of night-time collisions, when we only had GPS locations every 5 min, but no ACC data, we were able to determine the exact time of collision because the GPS locations remained from the time of collision at exactly the same place, where we later found the body.

We programmed the ACCs using different schedules depending on the specific objectives, but as a rule we set them to register 15-s activity bouts every 15 min between 05:00 and 21:00 UTC, with a byte count of 1188 and 16.7 Hz. We were able to distinguish 7 behaviours from the ACC graphs after 720 h field observations on 10 tracked birds from December 2018 to March 2019 and using these timed observations and the corresponding 2555 ground-truthed ACC sequences of known behaviours (see details in^114^) to train a model using the software Accelerater (^115,116^; http://smell.huji.ac.il/). Based on these data we could quantify the flight frequency of our birds, and using their GPS locations we could classify them as sedentary or migratory^91^, to check for sexual or migratory status-related differences in potential vulnerability to collision with aerial lines. Furthermore, we were able to determine whether collisions and road kills occurred during the day or by night. We defined nighttime as the period of full darkness (45 min after sunset to 45 min before sunset). All data were stored in Movebank (https://www.movebank.org/) and could be downloaded through the phone network thanks to a very good coverage in our study area, with no need to recapture the birds.

We defined the fate of each tracked bird (alive or dead) at the end of the study period (January 1st, 2023). We censored two birds whose loggers stopped working and one that lost the logger, respectively 28, 300 and 105 days before that end date, and considered the date of their last known location as the end of their monitoring period. The remaining 27 censored birds (Table 1) were alive at the end of the study period. We classified mortality events as caused by *anthropogenic* factors (collision with power or telephone line, collision with motor vehicle, collision with wire fence, predation by domestic cat) or *natural* factors (diseases, acute infectious processes, weakened body condition, or other cases where human infrastructure or any type of human involvement could be clearly discarded). The rapid location of the dead birds before they decompose thanks to daily reception of both, GPS data accumulated in the logger and ACC data on behavior helped us to identify the exact moment when the bird was dead, the exact place where the fatality occurred, and the activity and movements of the bird immediately before (e. g., whether it had flown across a power line or walked across a road). This information was decisive in determining the cause of death, which was later confirmed in all cases through necropsy by veterinarians at the University of Las Palmas de Gran Canaria. Mortality rates obtained from tagged individuals are not subject to the error sources that affect carcass searches, and do not need extrapolation to the entire range of the species.

### Cause-specific mortality analysis

We used Cox proportional hazards models, adjusted for staggered entry and right-censoring, to compare mortality risk among the various causes identified, and to assess the effects of multiple covariates on mortality. Survival time was the response variable, and we examined the effects of two covariates (*sex* and *migratory status* –sedentary or migratory-) on competing mortality risks, by using an extension to the Cox model that jointly considers the survival time and cause of death for an individual^75,117,118^. We used Akaike’s Information Criterion corrected for small sample size (AICc) for model selection and considered all models ≤ 2 ΔAICc competing^119^. Using this approach, we fit a Cox proportional hazard model considering two competing risks: *anthropogenic* (including all four types of collisions, i.e. with power lines, telephone lines, wire fences and vehicles) and *natural*, and included the two covariates mentioned above (sex and migratory status). A more detailed examination of differences among anthropogenic mortality causes was not possible due to small simple size. We obtained sex-specific estimates by producing Kaplan–Meier survival curves from fitted Cox proportional hazards models. We estimated anthropogenic and natural mortality rates considering that both are completely additive, i.e., that there are no compensatory processes acting on our study population (but see e.g.^120^). To estimate cause-specific mortality probabilities that accounted for each competing risk, we used nonparametric cumulative incidence functions implemented in R package ‘*cmprsk’*^121,122^. The analyses were carried out on R version 4.2.2^123^ using package ‘*survival*’^124^.

### Population simulation

Using Vortex 10^125^ we simulated how anthropogenic mortality affects the viability of Canarian houbaras, by examining the performance of the population under two scenarios, natural mortality only and natural plus anthropogenic mortality. We obtained annual mortality rates of male and female houbara bustards under both scenarios from our fitted Cox proportional hazards models, and reproductive rates from annual juvenile productivity surveys of the population carried out in May–June over seven consecutive years (2017–2023) in Lanzarote, and four years (2020–2023) in Fuerteventura. The surveys were conducted by two teams each of two people, operating from vehicles and provided with GPS, binoculars and 20–60 × telescopes. We distinguished males, females and first year birds, who by the time we did the surveys were dependent from her mother and formed a family unit generally isolated from other birds. At that time, most chicks have grown sufficiently large for the families to be detectable, but have not yet reached adult size, so that they can be unmistakably identified as young of the year^126^. More details of the survey methodology are given in Alonso et al.^95,126^). From the percentage of juveniles per 100 non-juvenile females and the number of offspring per female obtained in the surveys, we calculated the percentage of adult females breeding successfully each year as required by Vortex, i.e. those that had dependent young when we did the productivity survey. As adult females we considered those older than 2 years, using the age distribution calculated from our data in Vortex^125^, and guidelines in Lacy et al.^127^. The percentage of adult females breeding successfully obtained from surveys was compared with the value from 18 tagged females tracked over 3–4 years, and both values differed by only a 2.97%. The parameter values used in simulations are shown in Supplementary Table S1 online. We performed 100 iterations of each simulation over 50 and 100 years, and compared the population trajectories, probabilities of extinction, and final population sizes.

### Ethics statement

Capture, handling and marking houbara bustards were authorized and conducted under permissions issued by regional authorities (Viceconsejería de Medio Ambiente, Gobierno de Canarias, license 2015/10584). The weight of the logger plus harness material was below the commonly accepted limit of 5% of the weight of the birds. We did not observe any stress signs or behavioural alteration of the birds from marking. The experimental protocol was approved by the ethics committee of the Spanish National Research Council (CSIC) (https://www.csic.es/es/el-csic/etica/comite-de-etica-del-csic), and the methods used comply with the Spanish guidelines for ethical use in animal research (Spanish Committee for the Protection of Animals Used for Scientific Purposes, CEPAFIC, Royal Decree 53/2013 of the Spanish Ministry of the Presidence, BOE-A-2013-1337, https://www.boe.es/buscar/pdf/2013/BOE-A-2013-1337-consolidado.pdf). The study is reported in accordance with ARRIVE guidelines (https://arriveguidelines.org).

### Supplementary Information

Supplementary Information.

## Data Availability

The datasets generated during and/or analysed during the current study are available from the corresponding author on reasonable request.
