# Supplementary material for: Anthropogenic mortality threatens the survival of Canarian houbara bustards
Source: Sci Rep. 2024 Jan 24;14:2056. doi: 10.1038/s41598-024-52641-z (PMC10810086; doi:10.1038/s41598-024-52641-z)
Supplement: Supplementary file 1 — Supplementary Information. [file 41598_2024_52641_MOESM1_ESM.docx]

**Supplementary Information to:**

**Anthropogenic mortality threatens the survival of Canarian houbara bustards**

**Supplementary Table S1**. Parameters of Canarian houbara bustard used in Vortex 10 (Lacy & Pollack 2021) population models. Breeding data were measured by means of juvenile productivity surveys carried out in seven years (2017-2023), and confirmed with breeding success data from a sample of 18 marked females during six years (2019-2023). Survival data were obtained during this study (see details in Methods, section *Cause-specific mortality analysis*)

|  | **All islands** | | **Lanzarote** | | **Fuerteventura** | |  |
| --- | --- | --- | --- | --- | --- | --- | --- |
| **Vortex parameters** | **Natural mortality** | **Natural + anthropogenic mortality** | **Natural mortality** | **Natural + anthropogenic mortality** | **Natural mortality** | **Natural + anthropogenic mortality** |  |
| **Scenario Settings:** |  | | | | | |  |
| Number of iterations | 100 | | | | | | Default value in Vortex 10 (Lacy & Pollack 2021) |
| Numer of years (timesteps) | 50/100 | | | | | | Simulations were run for both periods |
| Number of populations | 1 | | | | | |  |
| **Species Description:** |  | | | | | |  |
| Inbreeding depression | yes | | | | | | Active by default in Vortex 10, see details in Lacy et al. (2021); for negative effects of inbreeding in houbaras, see Rabier et al. (2021) |
| Lethal equivalents | 6,29 | | | | | | Default value in Vortex 10 (Lacy & Pollack 2021) |
| **Reproductive System:** | polygynous | | | | | | Hingrat & Saint Jalme (2005), Hingrat et al. (2007) |
| Age of first offspring females | 2 | | | | | | Preston et al. (2015) |
| Maximum age of female reproduction | 15 | | | | | | Estimated, based on maximum longevity (23 years, Preston et al. 2015; 20 years, Dolman et al 2018), and senescence effects on breeding performance (Bacon 2017, Bacon et al. 2017) |
| Age of first offspring males | 2 | | | | | | Preston et al. (2015) |
| Maximum age of male reproduction | 15 | | | | | | Estimated, based on maximum longevity (23 years, Preston et al. 2015; 20 years, Dolman et al 2018), and senescence effects on breeding performance (Bacon 2017, Bacon et al. 2017) |
| Maximum lifespan | 15 | | | | | | 15 years in the wild (Bacon 2017, Bacon et al. 2017) (in captivity, 23 years, Preston et al. 2015; 32 years, Rabier et al. 2021) |
| Maximum no, of broods per year | 1 | | | | | | Own data |
| Maximum no. of progeny per brood | 3 | | | | | | Own data |
| Sex ratio at birth -in % males- | 50 | | | | | | Dolman et al. (2018) |
| **Reproductive Rates:** |  | | | | | |  |
| % adult females breeding | 15.55 | 15.55 | 16.01 | 16.01 | 7.91 | 7.91 | Based on juvenile productivity surveys 2017-2023 (Alonso et al. 2022, updated with 2023 data^1^); value confirmed with breeding success data from 18 marked females tracked over 3-4 years (own unpubl. data; only 2.97% difference between population value and marked sample value) |
| SD in % breeding due to EV | 6.9 | 6.9 | 6.88 | 6.88 | 13.49 | 13.49 |  |
| *Distribution of broods per year:* |  | | | | | |  |
| 0 broods | 0 | | | | | | Default value in Vortex 10 (Lacy & Pollack 2021) |
| 1 broods | 100 | | | | | | Default value in Vortex 10 (Lacy & Pollack 2021) |
| No. of offspring per female per brood: |  | | | | | |  |
| 1 offspring | 87 | | | | | | Based on juvenile productivity surveys 2017-2023 (Alonso et al. 2022, updated with data from 2023) |
| 2 offspring | 13 | | | | | |  |
| **Mortality rates:** |  | | | | | |  |
| *Mortality of females as %:* |  | | | | | |  |
| Mortality from age 0 to 1 | 9.82 | 15.6 | 9.82 | 15.6 | 9.82 | 15.6 | Based on juvenile survival estimated by Dolman et al. (2018); we assumed 50% higher mortality from age 0 in June (during productivity surveys, when juveniles are ca. 3-4 months old) to June age 1 than for ages >2 |
| SD in 0 to 1 mortality due to EV | 3 | 3 | 3 | 3 | 3 | 3 | Following Lacy et al. (2021) |
| Mortality from age 1 to 2 | 6.55 | 10.4 | 6.55 | 10.4 | 6.55 | 10.4 | This study; sex-specific mortality estimates by producing Kaplan-Meier survival curves from fitted Cox proportional hazards models |
| SD in 1 to 2 mortality due to EV | 2 | 2 | 2 | 2 | 2 | 2 |  |
| Annual mortality after age 2 | 6.55 | 10.4 | 6.55 | 10.4 | 6.55 | 10.4 |  |
| SD in mortality after age 2 | 2 | 2 | 2 | 2 | 2 | 2 |  |
| *Mortality of males as %:* |  |  |  |  |  |  |  |
| Mortality from age 0 to 1 | 11.10 | 23.1 | 11.10 | 23.1 | 11.10 | 23.1 | This study; assuming 50% higher mortality from age 0 in June (when juveniles are ca. 3-4 months old) to June age 1 (ca. 15-16 months old individuals) than for ages >2 |
| SD in 0 to 1 mortality due to EV | 3 | 3 | 3 | 3 | 3 | 3 | Following Lacy et al. (2021) |
| Mortality from age 1 to 2 | 7.40 | 15.4 | 7.40 | 15.4 | 7.40 | 15.4 | This study; sex-specific mortality estimates by producing Kaplan-Meier survival curves from fitted Cox proportional hazards models |
| SD in 1 to 2 mortality due to EV | 2 | 2 | 2 | 2 | 2 | 2 |  |
| Annual mortality after age 2 | 7.40 | 15.4 | 7.40 | 15.4 | 7.40 | 15.4 |  |
| SD in mortality after age 2 | 2 | 2 | 2 | 2 | 2 | 2 |  |
| **Catastrophes** |  | | | | | |  |
| Number of types of catastrophes | 1 | | | | | | Default value in Vortex 10 (Lacy & Pollack 2021) |
| *Frequency ans extent of occurence:* |  | | | | | |  |
| Local | yes | | | | | | Default value in Vortex 10 (Lacy & Pollack 2021) |
| Frequency % | 1 | | | | | | Default value in Vortex 10 (Lacy & Pollack 2021) |
| *Severity (proportion of normal values):* |  | | | | | |  |
| Reproduction | 0.5 | | | | | | Following guidelines in Lacy et al. (2021) |
| Survival | 1 | | | | | | Following guidelines in Lacy et al. (2021) |
| **Initial Population Size** | 577 | 577 | 452 | 452 | 109 | 109 | Ucero et al. (2021) |
| **Carrying Capacity (K)** | 3000 | 3000 | 1000 | 1000 | 2000 | 2000 | Assuming ca. 1000 for Lanzarote (846 km²) and 2000 for Fuerteventura (1660 km²) |
| SD in K due to EV | 300 | 300 | 100 | 100 | 200 | 200 | Following examples in Lacy et al. (2021, p. 60) |
| Future change in K? | no | | | | | | Default value in Vortex 10 (Lacy & Pollack 2021) |
| **Harvest** | no | | | | | | Houbara bustards are not hunted in the study area |
| **Supplementation** | no | | | | | | There is no supplementation in our study population |

^1^ Current annual juvenile productivity is 12.22 juveniles per 100 females (average for the last 7 years; Alonso et al. 2022, updated with data from 2023). Taking into account a sex ratio of 1.41 females per male (Alonso et al. 2020), this juvenile productivity is equivalent to 7.15 juveniles per 100 non-juvenile individuals of both sexes in the population.

**Supplementary Table S2**. Differences in survival estimates between sexes. The values were obtained from survival probability functions using ‘*survfit*’ of the “survival” package (Therneau & Lumley 2019). In bold, estimated annual survival values.

| Time (days) | both sexes | SE | females | SE | males | SE |
| --- | --- | --- | --- | --- | --- | --- |
| 46 | 0.980 | 0.019 |  |  | 0.967 | 0.033 |
| 147 | 0.960 | 0.028 |  |  | 0.932 | 0.046 |
| 179 | 0.940 | 0.033 | 0.952 | 0.046 |  |  |
| 184 | 0.920 | 0.038 |  |  | 0.898 | 0.056 |
| 318 | 0.900 | 0.042 | **0.905** | 0.064 |  |  |
| 321 | **0.880** | 0.046 |  |  | **0.863** | 0.064 |
| 435 | 0.860 | 0.049 |  |  | 0.829 | 0.070 |
| 536 | 0.840 | 0.052 | 0.857 | 0.076 |  |  |
| 629 | 0.820 | 0.054 | 0.810 | 0.086 |  |  |
| 657 | 0.800 | 0.057 |  |  | 0.794 | 0.075 |
| 666 | 0.780 | 0.059 |  |  | 0.760 | 0.079 |
| 686 | 0.760 | 0.060 |  |  | 0.725 | 0.083 |
| 687 | 0.740 | 0.062 |  |  | 0.690 | 0.086 |
| 864 | 0.720 | 0.064 |  |  | 0.654 | 0.089 |
| 1029 | 0.699 | 0.065 | 0.762 | 0.093 |  |  |
| 1067 | 0.679 | 0.066 |  |  | 0.618 | 0.091 |
| 1079 | 0.657 | 0.068 | 0.714 | 0.099 |  |  |
| 1323 | 0.625 | 0.072 |  |  | 0.566 | 0.097 |
| 1338 | 0.592 | 0.075 |  |  | 0.515 | 0.101 |
| 1408 | 0.557 | 0.078 | 0.625 | 0.120 |  |  |
| 1763 | 0.487 | 0.095 |  |  | 0.450 | 0.107 |

**Supplementary Table S3**. Differences in survival estimates between two scenarios: one in which all causes of natural and anthropogenic mortality are operating, and a natural mortality only scenario, in which anthropogenic mortality is suppressed. The values were obtained from survival probability functions using ‘*survfit*’ of the “survival” package (Therneau & Lumley 2019), including all birds (i.e., without covariates for sex and migratory status). In bold, annual survival values estimated from the model; underlined, survival estimates obtained for 365 days (1 year) from regression functions adjusted to the values provided by the model. Underlined, anual survival values obtained for exactly 365 days (1 year) from the regression functions adjusted to the survival values provided by the model. The difference between these values is 0.062, which is the approximate proportion of individuals died from anthropogenic causes in one year (see Fig. 3).

| Natural mortality only scenario | | | | | | |  | Natural and anthropogenic mortality scenario | | | | | | | |
| --- | --- | --- | --- | --- | --- | --- | --- | --- | --- | --- | --- | --- | --- | --- | --- |
| Time (days) | n.risk | n.event | survival  estimate | SE | lower 95% CI | upper 95% CI |  | Time (days) | n.risk | n.event | survival estimate | SE | lower 95% CI | upper 95% CI | |
| 46 | 51 | 1 | 0.980 | 0.019 | 0.943 | 1.000 |  | 46 | 51 | 1 | 0.980 | 0.019 | 0.943 | 1.000 | |
|  |  |  |  |  |  |  |  | 147 | 49 | 1 | 0.960 | 0.028 | 0.908 | 1.000 | |
| 179 | 48 | 1 | 0.960 | 0.028 | 0.907 | 1.000 |  | 179 | 48 | 1 | 0.940 | 0.033 | 0.877 | 1.000 | |
| 184 | 47 | 1 | **0.940** | **0.034** | **0.875** | **1.000** |  | 184 | 47 | 1 | 0.920 | 0.038 | 0.848 | 0.998 | |
|  |  |  |  |  |  |  |  | 318 | 46 | 1 | 0.900 | 0.042 | 0.821 | 0.987 | |
|  |  |  |  |  |  |  |  | 321 | 45 | 1 | **0.880** | **0.046** | **0.795** | **0.975** | |
| **365** |  |  | **0.936** |  |  |  |  | **365** |  |  | **0.874** |  |  |  | |
|  |  |  |  |  |  |  |  | 435 | 44 | 1 | 0.860 | 0.049 | 0.770 | 0.962 | |
| 536 | 43 | 1 | 0.918 | 0.039 | 0.843 | 0.998 |  | 536 | 43 | 1 | 0.840 | 0.052 | 0.745 | 0.948 | |
|  |  |  |  |  |  |  |  | 629 | 42 | 1 | 0.820 | 0.054 | 0.721 | 0.934 | |
| 657 | 41 | 1 | 0.895 | 0.044 | 0.812 | 0.987 |  | 657 | 41 | 1 | 0.800 | 0.057 | 0.697 | 0.919 | |
|  |  |  |  |  |  |  |  | 666 | 40 | 1 | 0.780 | 0.059 | 0.674 | 0.904 | |
|  |  |  |  |  |  |  |  | 686 | 39 | 1 | 0.760 | 0.060 | 0.651 | 0.888 | |
|  |  |  |  |  |  |  |  | 687 | 38 | 1 | 0.740 | 0.062 | 0.628 | 0.872 |  |
|  |  |  |  |  |  |  |  | 864 | 36 | 1 | 0.720 | 0.064 | 0.605 | 0.856 | |
| 1029 | 35 | 1 | 0.870 | 0.0500 | 0.777 | 0.973 |  | 1029 | 35 | 1 | 0.699 | 0.065 | 0.583 | 0.839 | |
|  |  |  |  |  |  |  |  | 1067 | 34 | 1 | 0.679 | 0.066 | 0.560 | 0.822 | |
|  |  |  |  |  |  |  |  | 1079 | 32 | 1 | 0.657 | 0.068 | 0.538 | 0.804 | |
| 1323 | 20 | 1 | 0.826 | 0.0636 | 0.710 | 0.973 |  | 1323 | 20 | 1 | 0.625 | 0.072 | 0.499 | 0.782 | |
| 1338 | 19 | 1 | 0.783 | 0.0737 | 0.651 | 0.941 |  | 1338 | 19 | 1 | 0.592 | 0.075 | 0.461 | 0.759 | |
|  |  |  |  |  |  |  |  | 1408 | 17 | 1 | 0.557 | 0.078 | 0.423 | 0.734 | |
|  |  |  |  |  |  |  |  | 1763 | 8 | 1 | 0.487 | 0.095 | 0.333 | 0.713 | |

**Supplementary Table S4**. Estimate of total houbara bustard deaths due to anthropogenic causes in the Canary Islands, considering an annual mortality rate of 6.38% due to these causes. In La Graciosa there are no power and telephone lines outside the only town on the island, and very few vehicles drive on the sand roads, generally at low speed; gtherefore, for that island we only estimated deaths due to cat predation, based on the cat predation rate found in the other two islands.

| Lanzarote: | | |  |  |
| --- | --- | --- | --- | --- |
| Houbara bustard census | mínimum | | 440 | 27.28 |
|  | máximum | | 452 | 28.02 |
| Fuerteventura: | | |  |  |
| Houbara bustard census | minimum | | 85 | 5.27 |
|  | maximum | | 109 | 6.76 |
| La Graciosa: | | |  |  |
| Houbara bustard census | | minimum | 12 | 0.06 |
|  | | maximum | 16 | 0.08 |
| Whole Canary Islands archipelago: | | |  |  |
|  | minimum | |  | 32.55 |
|  | maximum | |  | 34.78 |
|  |  | |  |  |


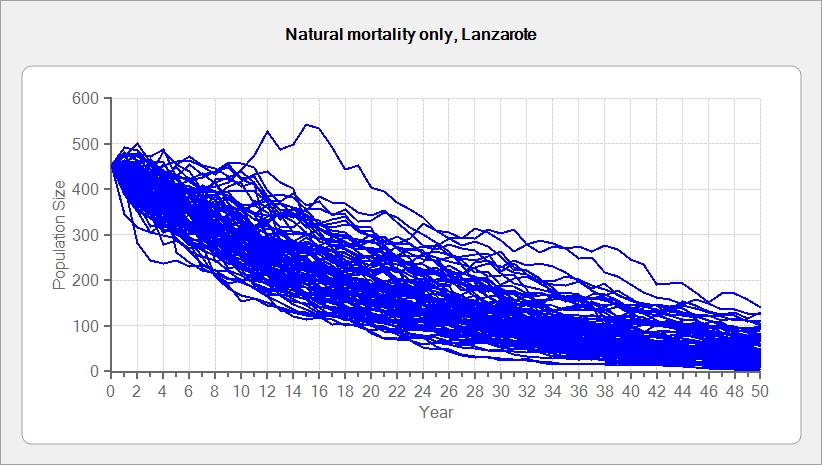

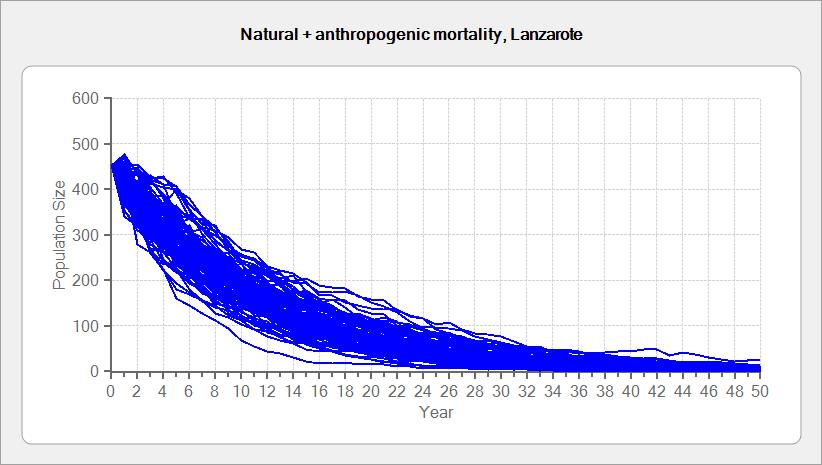

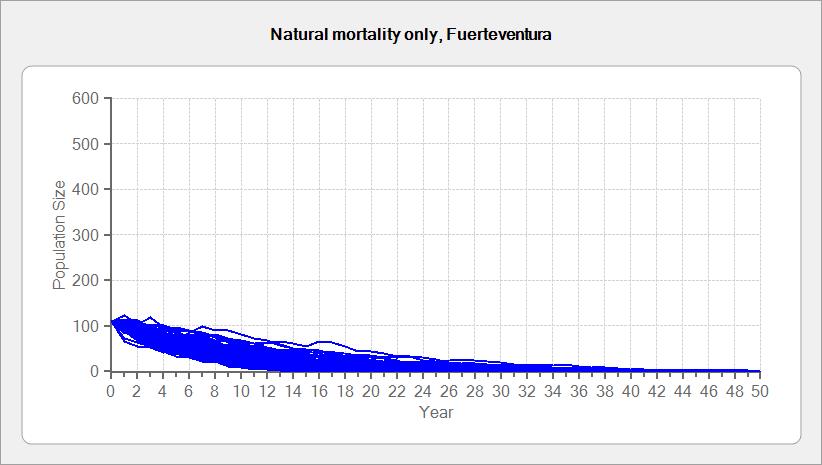

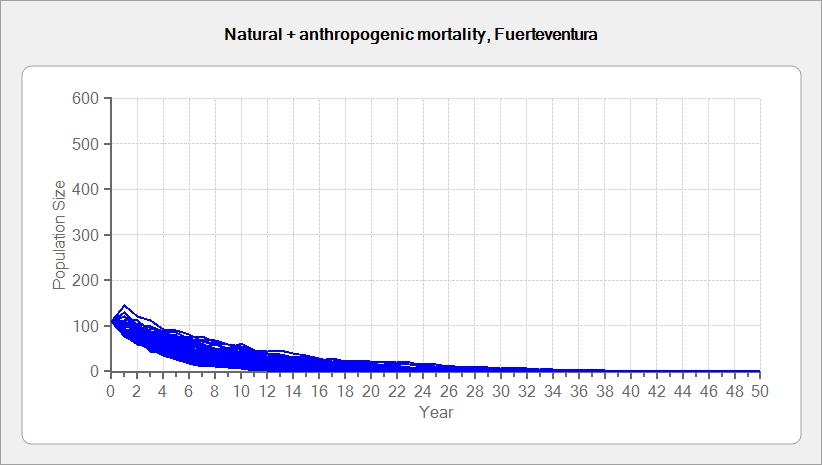

**Supplementary Figure S1**. Simulated trajectories of the houbara bustard populations in the two main islands where the species is present (Lanzarote, 452 individuals; Fuerteventura, 109 individuals) over 50 years under two scenarios, natural mortality only, and both natural and anthropogenic mortality. For each simulation, the exponential rate of increase (mean and SD), probability of extinction, final population size and mean genetic diversity (or expected heterozygosity) remaining in the extant population are given. The values for an extended simulation period of 100 years are also given.

**References**

Alonso, J. C., Ucero, A., Abril-Colón, I. & Palacín, C. Productividad juvenil en la avutarda hubara canaria (*Chlamydotis undulata fuertaventurae*). Unpublished report (Consejería de Transición Ecológica, Lucha Contra el Cambio Climático y Planificación Territorial, Gobierno de Canarias, 2022).

Bacon, L. Etude des paramètres de reproduction et de la dinamique d’une population renforcée d’outardes Houbara nord-africaines (Chlamydotis undulata undulata) au Maroc. PhD thesis. 192 pp (Muséum National d’Histoire Naturelle, 2017).

Bacon, L., Hingrat, Y. & Robert, A. Evidence of reproductive senescence of released individuals in a reinforced bird population. *Biol. Conserv*. **215**, 288–295 (2017).

Dolman, P. M., Collar, H. J. & Burnside, R. J. Captive breeding cannot sustain migratory Asian houbara *Chlamydotis macqueenii* without hunting controls. *Biol. Conserv*. **228**, 357-366 (2018).

Hingrat, Y. & Saint Jalme, M. Mating system of the Houbara Bustard *Chlamydotis undulata undulata* in eastern Morocco. *Ardeola* **52**, 91-102 (2005).

Hingrat, Y., Saint Jalme, M., Ysnel, F., Le Nuz, E. & Lacroix, F. Habitat use and mating system of the Houbara bustard in a semi-desertic area of North Africa: implications for conservation. *J. Ornithol*. **148** 39–52 (2007).

Lacy, R. C. & Pollak, J. P. Vortex: A Stochastic Simulation of the Extinction Process. Version 10.5.5. Chicago Zoological Society, Brookfield, Illinois, USA. (2021).

Lacy, R. C., Miller, P. S. & Traylor-Holzer, K. Vortex 10 User’s Manual. 30 March 2021 update. (IUCN SSC Conservation Planning Specialist Group & Chicago Zoological Society, Apple Valley, Minnesota, USA, 2021).

Preston, B. T., Saint Jalme, M., Hingrat, Y., Lacroix, F., Sorci, G. The sperm of aging male bustards retards their offspring's development. *Nat. Commun*. **6**, e6146 (2015).

Rabier, R., Lesobre, L. & Robert, A. Reproductive performance in houbara bustard is affected by the combined effects of age, inbreeding and number of generations in captivity. Sci Rep 11, 7813 (2021). <https://doi.org/10.1038/s41598-021-87436-z> (2021).

Therneau, T. M. & Lumley, T. Survival: survival analyses. R package version 2.44–1.1. Available from, <https://cran.r–project.org/web/> packages/survival/index.html. (2019).

(a)


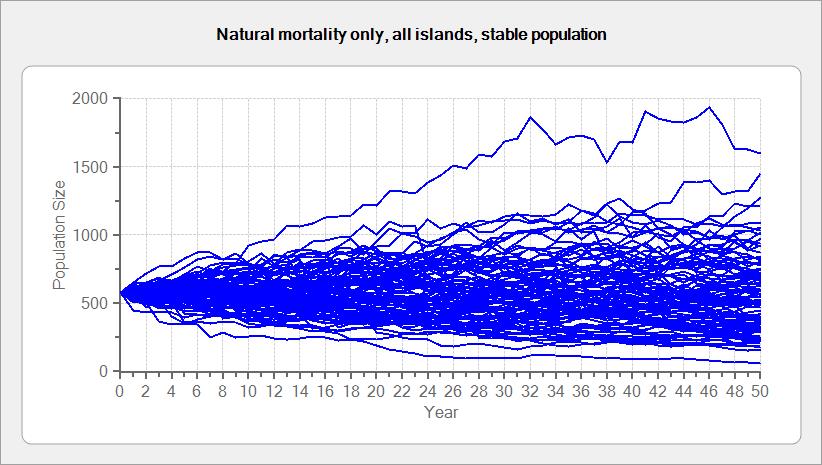

(b) (c)


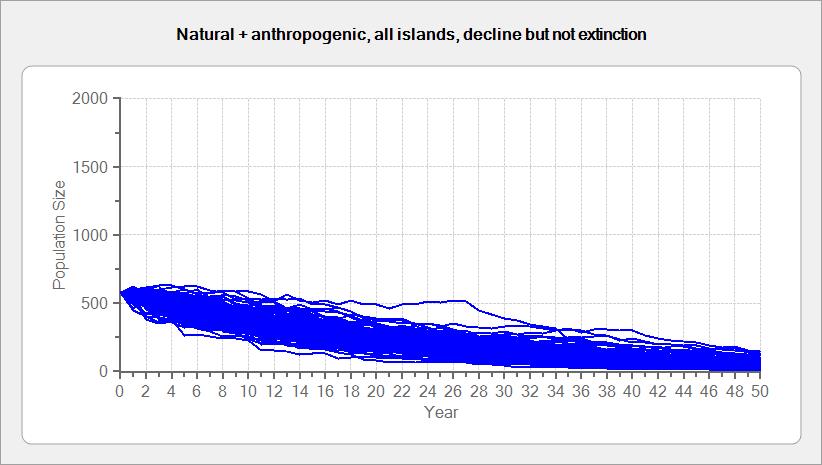

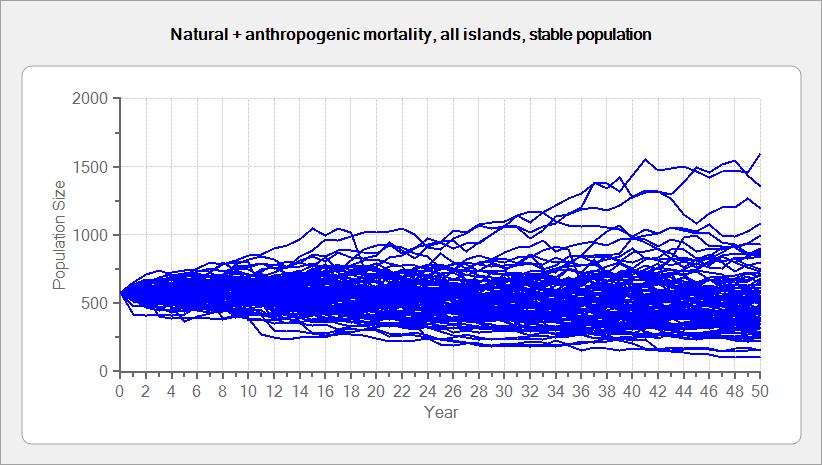

**Supplementary Figure S2**. Simulated trajectories of the houbara bustard population in the Canary Islands (initial population size = 577 birds) over 50 years under the following scenarios: (a) natural mortality only, and 23% adult females breeding successfully; (b) natural and anthropogenic mortality, and 23% adult females breeding successfully; (c) natural and anthropogenic mortality, and 32% adult females breeding successfully. For each simulation, the exponential rate of increase (mean and SD), probability of extinction, final population size and mean genetic diversity (or expected heterozygosity) remaining in the extant population are given. The population remains stable in simulations (a) and (c), and survives, although with a significant decrease, in simulation (b).
